# The potential of spectral domain optical coherence tomography imaging based retinal biomarkers

**DOI:** 10.1186/s40942-016-0054-7

**Published:** 2017-01-09

**Authors:** Prateep Phadikar, Sandeep Saxena, Surabhi Ruia, Timothy Y. Y. Lai, Carsten H. Meyer, Dean Eliott

**Affiliations:** 1 Department of Ophthalmology, King George’s Medical University, Lucknow, U.P. 226003 India; 2 Department of Ophthalmology and Visual Sciences, The Chinese University of Hong Kong, Shatin, Hong Kong; 3 Department of Ophthalmology, Pallas Klinik, Aarau, Switzerland; 4 Harvard Medical School, Massachusetts Eye and Ear, Boston, MA USA

**Keywords:** Age related macular degeneration, Biomarkers, Diabetic retinopathy, Inherited macular disorder, Optical coherence tomography, Retinitis pigmentosa, Vitreomacular interface disorders

## Abstract

**Background:**

Biomarker”, a merged word of “biological marker”, refers to a broad subcategory of medical signs that objectively indicate the state of health, and well-being of an individual. Biomarkers hold great promise for personalized medicine as information gained from diagnostic or progression markers can be used to tailor treatment to the individual for highly effective intervention in the disease process. Optical coherence tomography (OCT) has proved useful in identifying various biomarkers in ocular and systemic diseases.

**Main body:**

Spectral domain optical coherence tomography imaging-based biomarkers provide a valuable tool for detecting the earlier stages of the disease, tracking progression, and monitoring treatment response. The aim of this review article is to analyze various OCT based imaging biomarkers and their potential to be considered as surrogate endpoints for diabetic retinopathy, age related macular degeneration, retinitis pigmentosa and vitreomacular interface disorder. These OCT based surrogate markers have been classified as retinal structural alterations (macular central subfield thickness and cube average thickness); retinal ultrastructural alterations (disruption of external limiting membrane and ellipsoid zone, thinning of retinal nerve fiber layer and ganglion cell layer); intraretinal microangiopathic changes; choroidal surrogate endpoints; and vitreoretinal interface endpoints.

**Conclusion:**

OCT technology is changing very quickly and throughout this review there are some of the multiple possibilities that OCT based imaging biomarkers will be more useful in the near future for diagnosis, prognosticating disease progression and as endpoint in clinical trials.

## Background

“Biomarker”, a merged word of “biological marker”, refers to a broad subcategory of medical signs that objectively indicate the state of health, and well-being of an individual. These can be anatomical, biochemical, molecular parameters or imaging features. They are measurable by physical examination, laboratory assay or medical imaging. In clinical practice, they are useful in refinement of diagnosis, measuring disease progression or predicting and monitoring effects of therapeutic interventions. Their source can be body fluid such as plasma, urine, synovial fluid or tissue biopsy [1]. There are clear potential benefits in using biomarkers. Information can be obtained earlier, more quickly, and more economically.

Imaging biomarkers target the diseased organ or tissue and are hence specific indicators. Biochemical biomarkers in contrast, tend to integrate information from the entire body. Ultimately, biomarkers can be used to detect a change in the physiologic state of a patient that correlates with the risk or progression of a disease or with the susceptibility of a disease to a given treatment. Biomarkers hold great promise for personalized medicine as information gained from diagnostic or progression markers can be used to tailor treatment to the individual for highly effective intervention in the disease process.

## Biomarkers as surrogate endpoints

Biomarkers are often used as surrogate endpoints in clinical trials. A surrogate endpoint has been defined as ‘a biomarker intended to substitute for a clinical endpoint’, the latter being ‘a characteristic or variable that reflects how a patient feels, functions, or survives’ [2]. Clinical endpoints are variables that represent a study subject’s health and wellbeing from the subject’s perspective. These endpoints have the potential to definitively demonstrate whether interventions in a trial are effective or ineffective, as well as safe or unsafe. Any measurement short of the actual outcome could be regarded as a surrogate endpoint biomarker. However, although all surrogate endpoints are biomarkers, not all biomarkers are useful surrogate endpoints. The ideal biomarker is one through which the disease comes about or through which an intervention alters the disease [3]. In looking for criteria for deciding which biomarkers are good candidates for surrogate endpoints we can turn to the guidelines that Austin Bradford Hill propounded for helping to analyze association in determining causation [4]. To be considered as a surrogate endpoint, there must be solid scientific evidence (epidemiologic, therapeutic, and/or pathophysiologic) that a biomarker consistently and accurately predicts a clinical outcome. This requires the determination of relevance and validity. Relevance refers to a biomarker’s ability to appropriately provide clinically relevant information to the public, the healthcare providers, or health policy officials. Validity refers to the need to characterize a biomarker’s effectiveness or utility as a surrogate endpoint. The biomarker proposed as a surrogate should be capable of being measured objectively, accurately, precisely and reproducibly. Biomarkers are also important in the development of new drug therapies through identification of drug targets [5]. They also serve as “progression” markers to delineate the development and course of a disease. The changes in these progression markers can be used to understand the effect of therapy in altering the disease process.

Optical coherence tomography (OCT) is a reliable, quick, sensitive, non-invasive, user-friendly device that provides high-resolution in vivo imaging of retinal microstructures. OCT based surrogate endpoints have proved useful to identify and study the disease process (diagnostic, prognostic and in clinical trial) in various ocular disorders.

The aim of this review article is to analyze various OCT based imaging biomarkers and their potential to be considered as surrogate endpoints for diabetic retinopathy (DR), age related macular degeneration (AMD), retinitis pigmentosa (RP) and vitreomacular interface (VMI) disorder. These OCT based surrogate markers have been classified as retinal structural alterations [macular central subfield thickness (CST) and cube average thickness (CAT)]; retinal ultrastructural alterations [disruption of external limiting membrane (ELM) and ellipsoid zone (EZ), thinning of retinal nerve fiber layer (RNFL) and ganglion cell layer (GCL)]; choroidal surrogate endpoints; and vitreoretinal interface endpoints.

## Biomarkers in diabetic retinopathy

Diabetic retinopathy (DR) is characterized by microaneurysms, capillary nonperfusion, and ischemia within the retina, ultimately leading to neovascularization and/or macular edema. Diagnosis is mostly based on fundus examination and fundus florescence angiography. But SD-OCT based biomarkers helps us to identify the ultrastructural alterations in retina even in early phases of the disease and their gradation increases with severity of DR. These biomarkers are also useful to evaluate the response to therapy and modify our treatment protocol accordingly. Thus these biomarkers serve as an endpoint in clinical trial.

### Structural alterations

SD-OCT based macular CST and CAT provide reliable objective standard estimates for screening of diabetic macular edema [6]. Several studies have correlated OCT based retinal thickness with visual acuity in diabetic macular edema [7–10]. We observed an increase in CST and CAT on SD-OCT with increased severity of retinopathy. CST and CAT serve as surrogate markers for prognosticating the disease severity. Targeted screening of diabetic macular edema, in a population, by these imaging biomarkers serve as a significant indicator for progression of disease process within the grade of retinopathy, which may not be evident clinically.

Disorganization of the foveal retinal inner layers and photoreceptor ELM disruption have been documented as robust SD-OCT based imaging biomarkers for predicting visual outcome in eyes with center involving diabetic macular edema. Investigation shows that disorganization of the retinal inner layers seems to be correlated with current visual acuity in individuals with existing or resolved centres involved DME. Disorganization of the retinal inner layers affecting 50% or more of the central 1-mm-wide zone centered on the fovea is associated with worse visual acuity. This holds true even in eyes with reduced vision despite edema resolution or, conversely, in eyes with good vision despite concurrent edema [11].

### Ultrastructural alterations

**Fig. 1:**
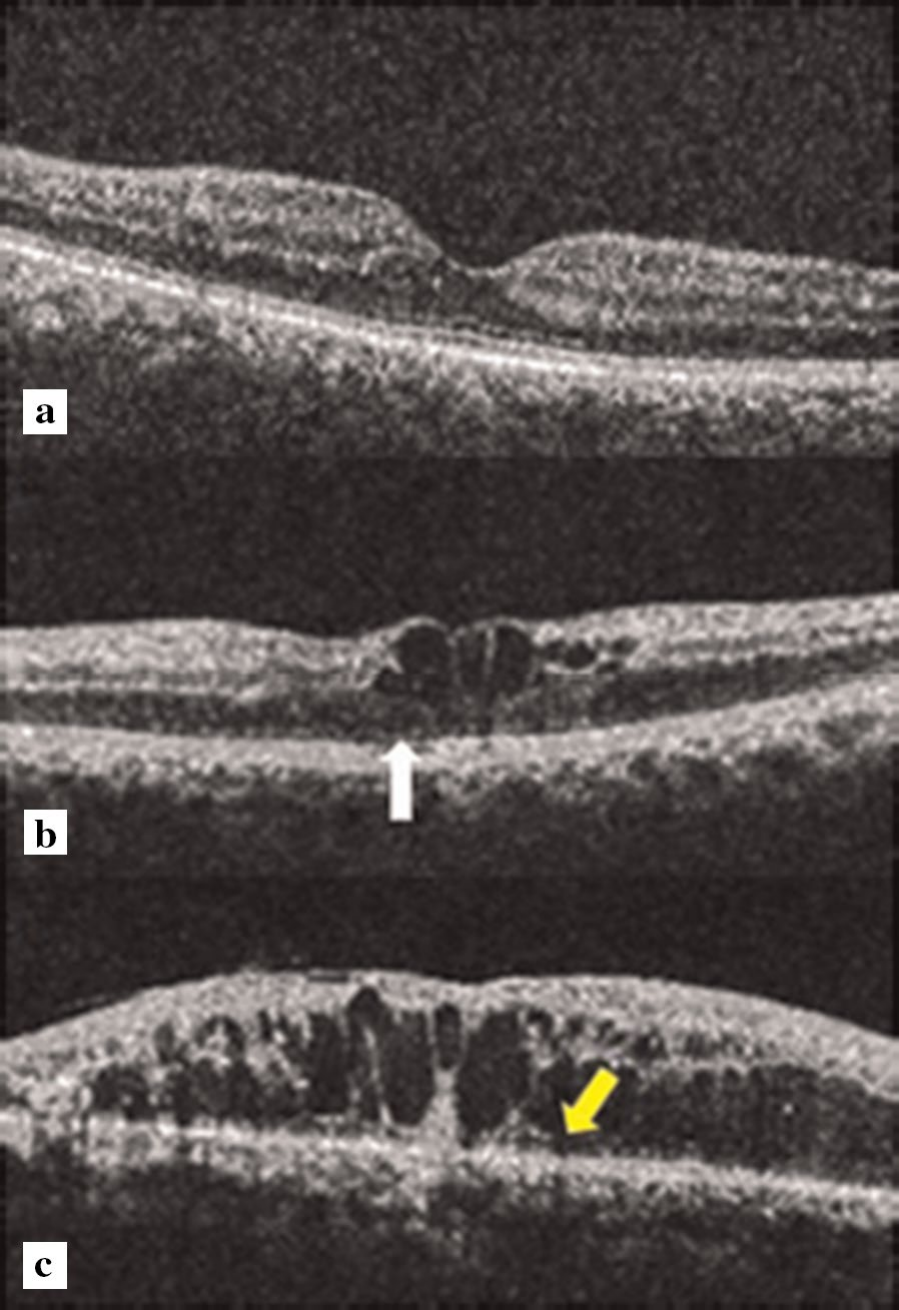
Spectral domain optical coherence tomography showing grades of disruption of the ELM and EZ. **a** Grade 0: no disruption of ELM and EZ. **b** Grade 1: ELM disruption (*white arrowhead*), EZ intact. **c** Grade 2: both ELM and EZ disrupted (*yellow arrow*)

Retinal photoreceptor ELM and EZ disruption grading systems [12] may serve as surrogate biomarkers in determining the progression of disease. Progression of structural alterations with severity of diabetic retinopathy has been graded in our earlier studies. Grade 0 no disruption of ELM and EZ; grade1 ELM disruption but intact EZ; grade 2 both ELM and EZ disruption [13] (Fig. 1). These grades co-relate with log mar visual acuity. It was also showed for the first time that ELM disruption occurred earlier than disruption of the EZ. This was based on the observation that the ELM has tight junctions similar to those between retinal pigment epithelium (RPE) cells. Therefore, the ELM acts like the third outer blood retinal barrier and its disruption contributes to fluid accumulation in diabetic macular edema. The disruption of the EZ is secondary to disrupted ELM. These classification systems provide a systematic approach to the diagnosis and management of diabetic macular edema and are useful for execution and analysis of clinical studies [14].

**Fig. 2:**
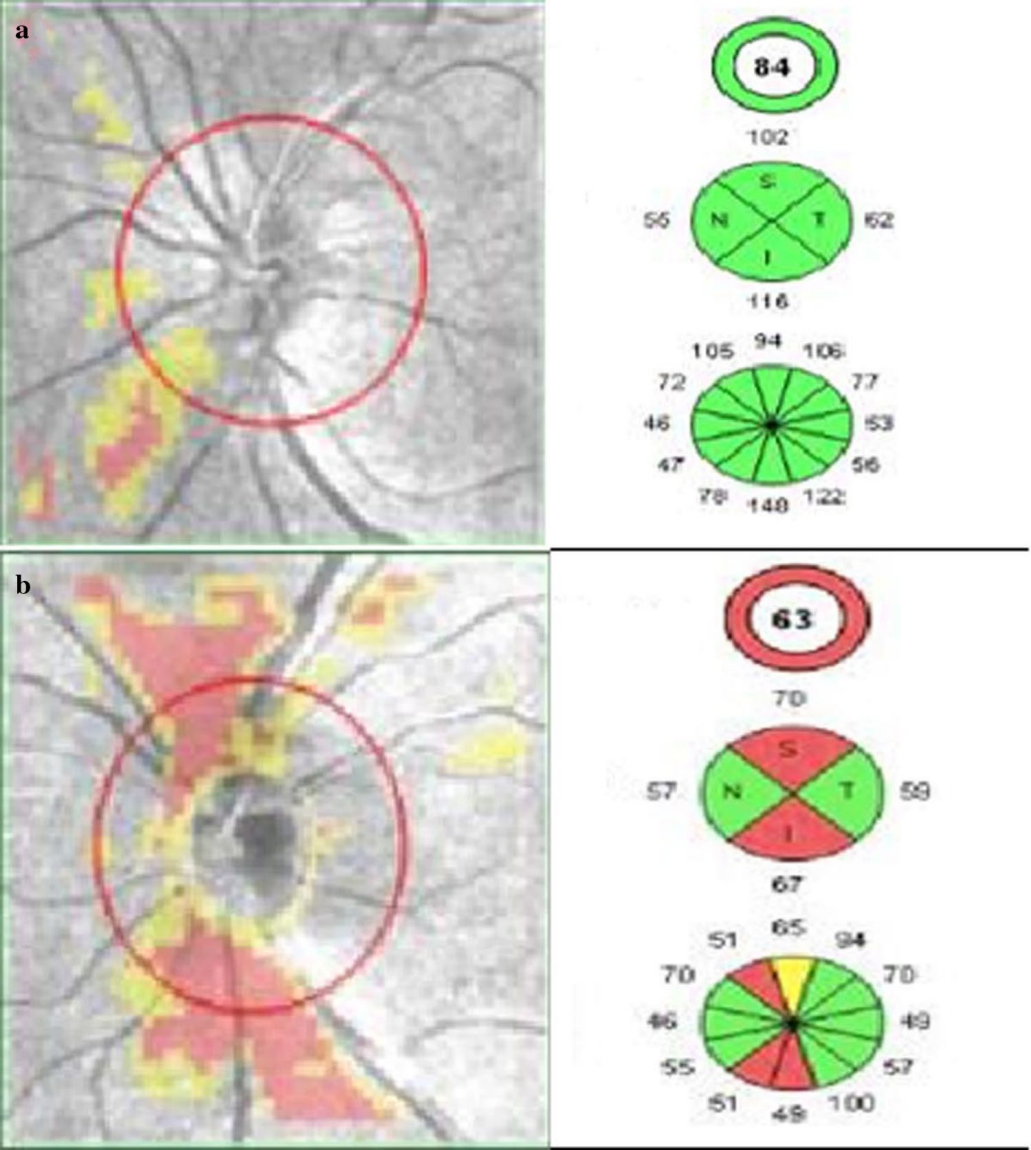
Retinal Nerve Fiber Layer (RNFL) thickness analysis using optic disc cube 200 × 200 feature depicting on RNFL thickness deviation map **a** left eye of patient with non-proliferative diabetic retinopathy showing RNFL thinning, **b** left eye of patient with proliferative diabetic retinopathy showing thinning of RNFL

**Fig. 3:**
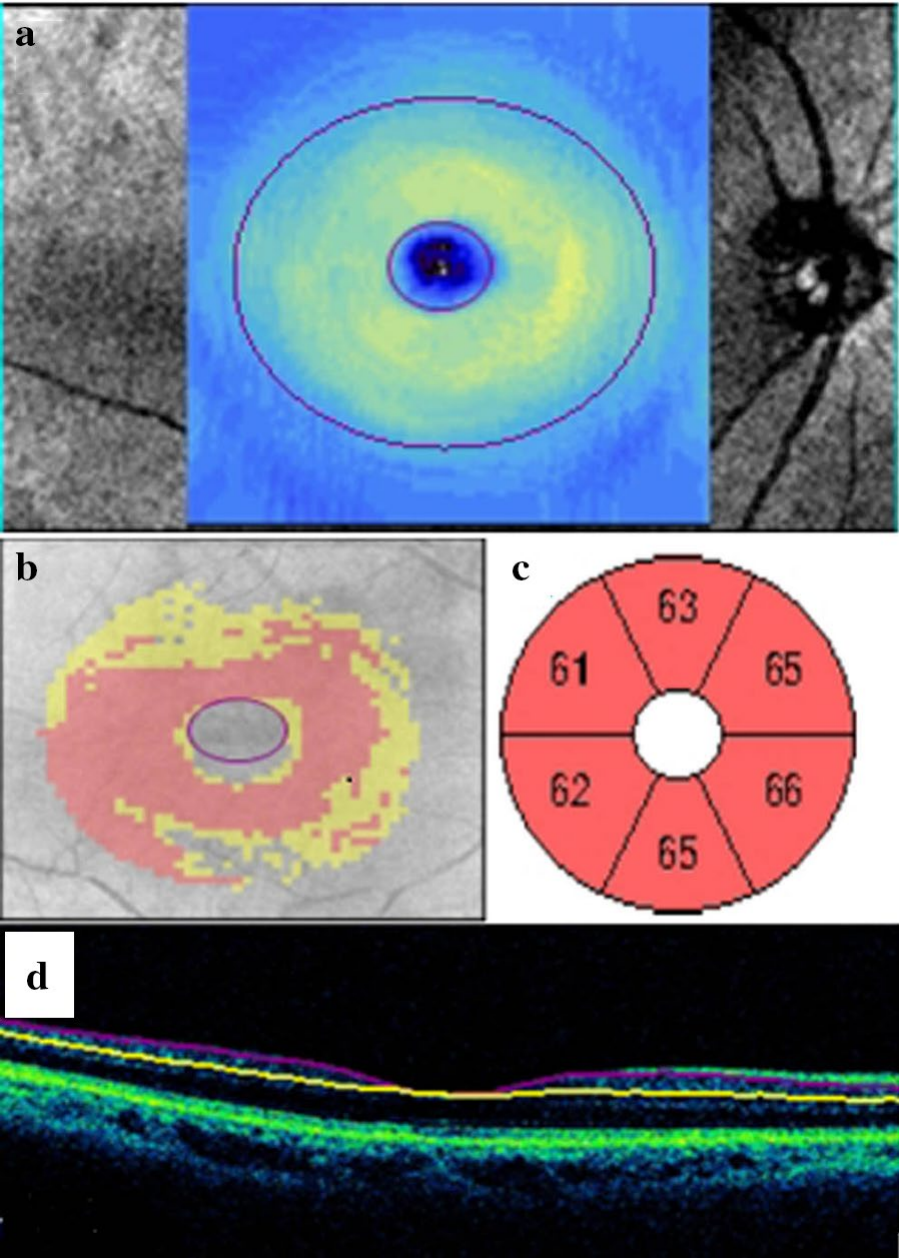
Ganglion Cell Analysis in a patient of diabetes showing thinning in thickness of GCL in GCL thickness map (**a**), deviation map (**b**), GCL sectoral quantative analysis (**c**). **d** Cross-sectional retinal imaging of GCL (layer)

Various studies showed a correlation of RNFL thinning with severity of type 2 DR on SD-OCT [15]. Significant decrease in RNFL thickness was observed with increase in the severity of DR (Fig. 2). RNFL thinning is associated with progression of DR and poor glycemic control [16, 17]. Rodrigues et al. [18] reported that neuroretinal changes precede vascular signs in diabetes mellitus. They observed a significant thinning of GCL and RFNL in patients with diabetes mellitus with no DR (Fig. 3).

A new parameter, “parallelism,” has been projected to evaluate retinal layer integrity using SD-OCT. OCT images are skeletonized and the orientation of segmented lines in the image is termed “parallelism”. The orientation of photoreceptor layer status at the fovea has been categorized, including continuity of the ELM, inner segment EZ, and the presence of hyperreflective foci in the outer retinal layers. Parallelism was observed to be significantly lower in eyes with diabetic macular edema in comparison to normal eyes. A positive correlation with visual acuity was also documented. Eyes with an intact EZ or ELM had significantly better visual acuity and higher parallelism than eyes with a discontinuous or absent EZ or ELM. Significantly higher parallelism and better visual acuity was observed in the group without hyperreflective foci in the outer retinal layers. This novel image parameter ‘parallelism’ serves as a potential biomarker to prognosticate visual outcome in diabetic macular edema [19].

### Choroidal surrogate endpoints

Choroidal thickness can be measured using SD-OCT high-definition raster scans in the majority of diabetic eyes. Choroidal thickness is altered in diabetes and related to the degree of severity of retinopathy [20, 21]. Presence of diabetic macular edema is associated with a significant decrease in the choroidal thickness. Regatieri et al. [22] observed that the mean subfoveal choroidal thickness was thinner in patients with diabetic macular edema or treated proliferative diabetic retinopathy, compared with normal subjects. Choroidal morphological features are altered in patients with moderate to severe DR [23].

## Biomarkers in age related macular degeneration

Age related macular degeneration (AMD) is a progressive degenerative disorder leading to gradual deterioration of central vision. One of the early clinical features in AMD is the appearance of drusen. On SD-OCT, drusen are defined by an elevation of the overlaying RPE above a certain threshold. One of the advantages of using SD-OCT imaging for measuring and following drusen over time is the capability of capturing the two and three dimensional features of drusen using cross-sectional B-scans, Enface topographical maps, and drusen volume and area measurements. When using cross-sectional B-scans, the integrity of the RPE and the photoreceptors overlying drusen can be visualized in great detail. SD-OCT images can show structural changes predictive of disease progression to late AMD, such as the intra-or subretinal fluid indicative of neovascular AMD [24], hyper reflective foci overlying drusen, subsidence of the outer retina, and heterogenous internal hyper reflectivity of drusenoid lesions indicative of nascent geographical atrophy [25], and choroidal thickness measurements below drusen of <135 µm [26], which is indicative of evolving geographical atrophy.

### Structural alteration

SD-OCT imaging has the advantage that it can measure changes in drusen volume, which is a far more sensitive technique for measuring changes in drusen size compared with area measurement. The reason why drusen volume was found to be a more sensitive indicator of drusen growth compared with area measurements is because area measurements tended to plateau while drusen volume continued to increase over time. Folgar et al. measured retinal pigment epithelium-drusen complex (RPEDC) volume to predict progression of intermediate AMD. Greater baseline OCT drusen volume was associated with progression to choroidal neovascularisation. Greater baseline RPEDC abnormal thinning volume was associated with significant increase in RPEDC abnormal thinning volume, and progression to central and non-central geographical atrophy [27].

The FDA approved, commercially available, and fully automated SD-OCT drusen segmentation algorithm offers an accurate, reliable, and standardized method for following drusen morphology over time [28–31]. A drusen baseline volume of 0.03 mm^3^ has been shown to be suitable to follow drusen growth [32] and the cube-root strategy should be used to evaluate drusen growth/shrinkage over time [33].

Ever since OCT became available, a huge effort has been made to identify OCT biomarkers that facilitate neovascular age related macular degeneration (nAMD) management and provide solid surrogate variables for treatment response and functional prognosis [34]. Three pathologic changes affecting central retinal morphology have been described in nAMD patients; intraretinal cystoid fluid, subretinal fluid, and pigment epithelial detachment [35, 36]. The presence of exudative cystoid fluid is an important finding on OCT as cysts are associated with a higher risk for visual loss associated with fibrosis and atrophy [37]. Therefore, intraretinal cystoid fluid is considered the most relevant prognostic biomarker in nAMD [38]. In end stage AMD, intraretinal cystoid fluid may be present above the atrophic scar, which appears as a hyperreflective and thickened RPE on OCT. The presence of degenerative cystoid fluid and an underlying fibrotic scar are thought to be irreversible and patients may not benefit from further anti-VEGF therapy.

Despite its initial popularity functional outcomes correlate poorly with central retinal thickness. Solely relying on CRT to make clinical decisions or as retreatment criteria in clinical trials is not recommended [39, 40]. However, central retinal thickness gives a first impression of retinal topography [41].

The optical density ratio (ODR) might be a valuable biomarker in nAMD as it correlates well with BCVA under anti-VEGF therapy and may be useful for differentiation as well as prognosis [42]. ODR compares the optical density of fluid accumulation in or under the retina to the optical density of the vitreous body. Optical density ratios change in the course of the disease because the blood retinal barrier regains function under anti-VEGF therapy and prevents the choroidal neovascularisation from leaking. A high optical density signal indicates increased reflectivity of the fluid accumulation, which is assumed to be caused by the protein concentration in the subretinal fluid [43] and is therefore thought to be a direct indicator for the blood-retinal barrier function [44]. Further Ahlers et al. showed that ODR changes correlate well with visual acuity changes under anti-VEGF therapy. Studies with larger sample sizes and longer follow-up are however needed to determine sensitivity and specificity for clinical use.

### Ultrastructural alterations

Drusen and intraretinal migration of retinal pigment epithelium have been associated with hyperreflective foci (HF) detected by SD-OCT. Proliferation and inner retinal migration of HF occurred during follow-up in eyes with intermediate AMD has been observed. HF proliferation and migration serve as biomarkers for progression of geographic atrophy [45].

External limiting membrane together with ellipsoid zone is considered a criterion that directly reflects photoreceptor function [46]. However, ELM is no predictor for individual loss or recovery in BCVA, but rather mirrors the current functional state of the retina [47].

It has been shown histologically that photoreceptors overlying drusen undergo degeneration. SD-OCT and adaptive optics has been used to monitor drusens over time for their progression in terms of size and their direct effect on the overlying photoreceptors [48]. With the use of microperimetry, functional data of photoreceptors can be obtained. These qualitative imaging-based biomarkers provide a valuable tool for detecting the earlier stages of the disease, tracking progression, and monitoring treatment response.

### Choroidal surrogate endpoints

There is thickening of choroid in the eyes with polypoidal choroidal vasculopathy (PCV) [49, 50]. A significant reduction in subfoveal choroidal thickness is noted after anti-VEGF therapy in AMD and PCV [51, 52]. As stated earlier choroidal thickness measurements below drusen of <135 µm [26], is indicative of evolving geographical atrophy.

## Biomarkers in retinitis pigmentosa and other inherited macular disorders

### Structural alteration

SD-OCT line scans serve as tool for structural biomarkers and full-field standard automated perimetry serve as functional biomarkers in patients with autosomal dominant retinitis pigmentosa. The total photoreceptor layer as well as the photoreceptor EZ width have been documented to have a significant correlation with functional biomarker of visual sensitivity obtained on automated perimetry [53].

### Ultrastructural alteration

The edge of the EZ in patients with Retinitis Pigmentosa indicates a transition zone between relatively healthy and relatively degenerate retina. Birch et al. [54] reported that the EZ provides a sensitive biomarker for progression in retinitis pigmentosa. They also suggested that OCT identification of the EZ in each patient may allow for the design of patient-specific visual fields to monitor disease progression in clinical trials [55].

SD-OCT images of patients suffering from inherited macular diseases can be of value to assess the integrity of the photoreceptor layer. Giannini et al. showed that texture analysis was valuable to characterize the structure and texture of the regular horizontal stratification of the photoreceptor layer in SD-OCT images. This method was highly sensitive for assessing the pathological changes of the ellipsoid zone in patients compared with age-matched controls [56].

**Fig. 4:**
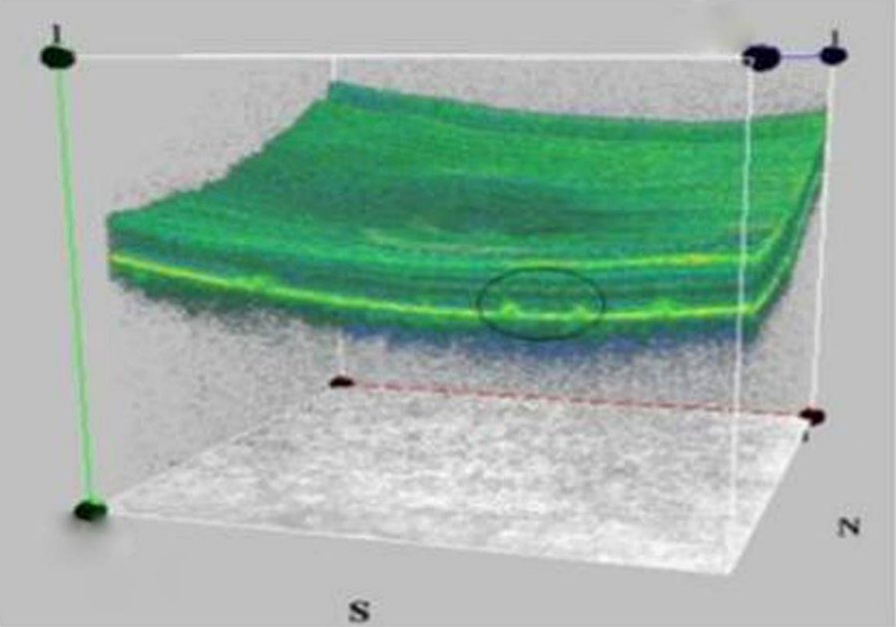
Three dimensional visualization shows flecks at the level of retinal pigment epithelium (enclosed in *black circle*)

Stargardt disease is an autosomal recessive macular dystrophy, linked to mutation of ABCA4 gene, characterized by early onset, rapid progression and poor visual outcome. Mutation in ABCA4 results in abnormal accumulation in RPE and consequent RPE degeneration and photoreceptor disruption. This results in macular atrophy and fleck like deposits in the retina of varying size and shapes. SD-OCT is helpful in these cases in revealing photoreceptor disruption and appropriate localization of the flecks in different layers of the retina and their anatomic configuration with one another [57]. SD-OCT can identify thickening and increased hyperreflectivity of the external limiting membrane and can serve as possible transient biomarker of early Stargardt disease [58]. Three-dimensional SD-OCT imaging provided novel findings showing presence of hyper-reflective flecks not only at the level of RPE and ONL but also at sub-RPE level in the case of stargardt disease [59] (Fig. 4).

## Biomarkers in disorders of the vitreomacular interface

Optical coherence tomography is the gold standard for the diagnosis and management of vitreomacular interface (VMI) diseases [60, 61]. Clinical biomicroscopic examination and other imaging modalities are limited in their capabilities to fully diagnose and document diseases of the VMI as vitreous membranes are often clinically invisible. SD-OCT is clinically useful to determine if a posterior vitreous detachment is complete, which may inform the management of VMI diseases. The panel of vitreoretinal disease experts provided anatomic definitions and classification of vitreomacular adhesion (VMA), vitreomacular traction (VMT) and full thickness macular hole (FTMH) [62].

### Vitreoretinal interface endpoints

**Fig. 5:**
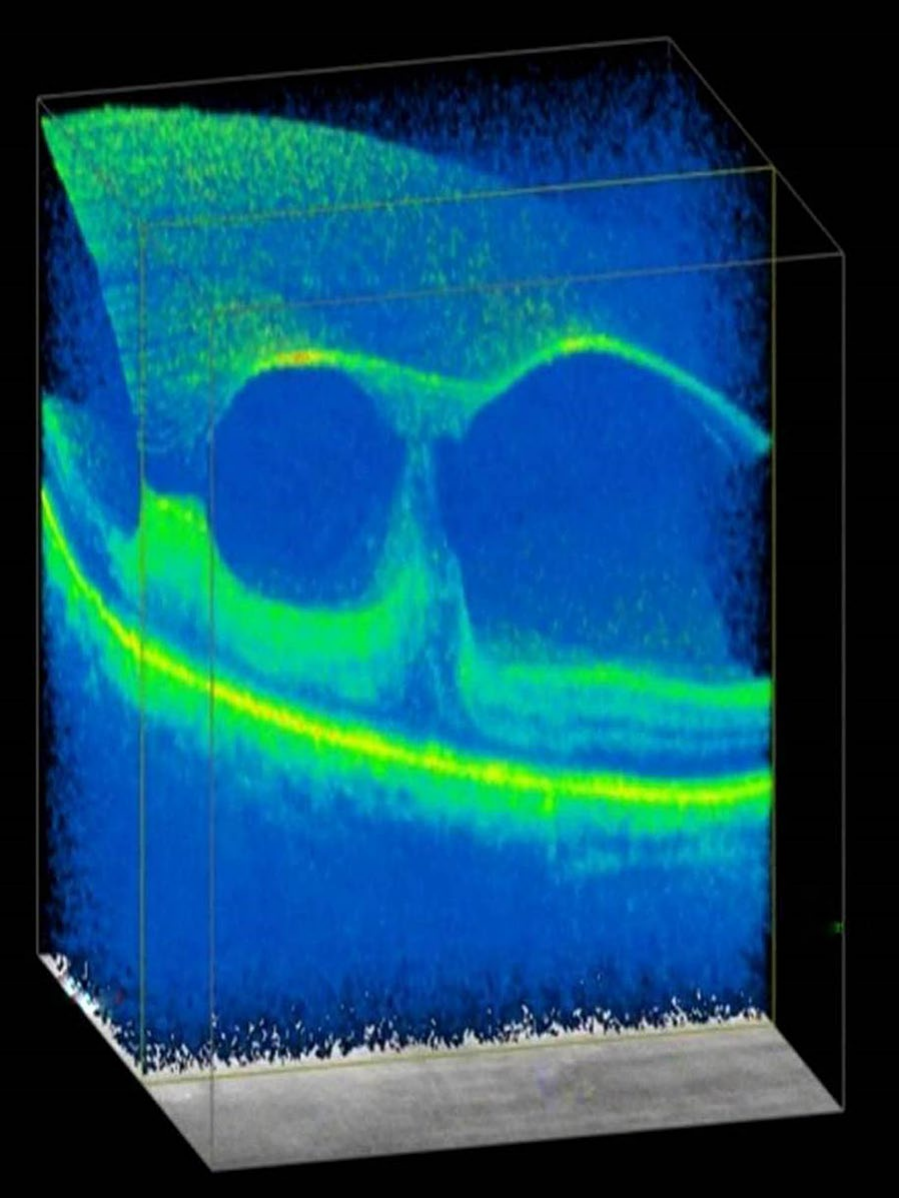
Three dimensional imaging along the X plane showing vitreo-retinal interaction with elevation of inner retinal layers at sites of persistent attachment of vitreous

Three dimensional imaging along with segmentation techniques provided comprehensive evaluation of the surface topography as well as foveal and extrafoveal anatomical configuration of vitreomacular affection [63] (Fig. 5). VMT can be subclassified by the diameter of vitreous attachment to the macular surface as measured by OCT, with attachment of 1500 μm or less defined as focal and attachment of more than 1500 μm as broad. When associated with other macular disease, VMT is classified as concurrent. SD-OCT provides high-resolution images to judge whether VMA is isolated or concurrent. OCT can also be used to sequentially follow VMT over time to detect resolution of the traction or, in some cases, progression to FTMH. OCT is necessary for an accurate diagnosis and guides preoperative decision-making and surgical planning [64]. SD-OCT has also been helpful to explain patient’s symptoms post-ocriplasmin [65].

### Epiretinal membrane

There is a wide range of pathology from epiretinal membranes (ERMs) that can be visualized by SD-OCT.

### Structural alterations

A thin ERM may cause minimal alteration of the underlying retinal architecture with only a slight change in the foveal contour. However, ERMs can also result in a complete loss of the foveal contour with cystoid intraretinal fluid and marked thickening of the retina. More subtle findings on SD-OCT can include hypo-reflective cystic spaces visualized between the ERM and the internal limiting membranes. The thickness of the ERM and degree of macular distortion and pseudocyst formation can help prognosticate the benefit of vitrectromy with membrane peel or prompt investigation of other causes for the patient’s symptoms.

### Ultrastructural alteration

SD-OCT is useful to predict the outcome after ERM peeling. Multiple studies of eyes with ERM have demonstrated that the preoperative integrity of the EZ on SD-OCT helps prognosticate postoperative visual acuity [66–68].

“Parallelism,” has also been projected to evaluate retinal layer integrity in individuals with epiretinal membrane. The more parallel the retinal layers, the better the visual acuity. Parallelism is also documented to significantly correlate with metamorphosia [69].

## Future prospectives

With advanced OCT systems commercially available, the great challenge is to find ways to enhance tissue contrast or add functional tools to obtain more information in addition to the recording of morphological structures and thus extend the clinical applicability of OCT. Functional approaches are of great interest as early diagnosis of retinal changes is known to be of vital importance because structural pathologies might be linked to irreversible damage and visual loss. Doppler OCT and polarization-sensitive OCT are currently most commonly used in retinal studies. These systems measure functional variables such as blood flow and velocity, as well as enhanced tissue contrast. *Spectroscopic OCT* is a functional extension of OCT, allowing for example oxygen measurements in combination with OCT measurements [70, 71]. As changes in oxygen consumption are associated with changes in various diseases, including AMD [72], it could facilitate future oxygen measurements and help diagnose retinal diseases earlier. *Photoacoustic tomography* could enable oxygen saturation mapping and high-resolution visualization of retinal and choroidal vascularization [73, 74]. The current gold standard when it comes to functional measurements of the retina is electrophysiology. It is time consuming and has limited depth resolution. *Optophysiology* is an OCT-based, contact-free technique that allows optical imaging of retinal responses to stimuli such as light flickering [75, 76].

## Conclusion

OCT based imaging biomarkers helps us to pick up disease at an early stage, to confirm our diagnosis in case of dice situation, grade the severity of disease (both qualitatively and quantitatively) and to modify our treatment regimen accordingly. To conclude CST and CAT are increased in diabetic macular edema. Significant decrease in RNFL, GCL and choroidal thickness is associated with increase in the severity of DR. SD-OCT imaging can be used for measuring and following drusen over time. Hyper reflective foci overlying drusen, subsidence of the outer retina, and heterogenous internal hyper reflectivity of drusenoid lesions is indicative of nascent geographical atrophy, and decrease in choroidal thickness is indicative of evolving geographical atrophy. Drusen volume can predict progression of intermediate AMD. Three pathologic changes affecting central retinal morphology in neovascular AMD patients are; intraretinal cystoid fluid, subretinal fluid, and pigment epithelial detachment. The optical density ratio (ODR) is a valuable biomarker in nAMD and is thought to be a direct indicator for the blood-retinal barrier function. Increased thickness of choroid is seen in eyes with PCV. External limiting membrane (ELM) together with ellipsoid zone and microperimetry directly reflects photoreceptor function. Disorganization of the foveal retinal inner layers and photoreceptor EZ/ELM disruption have been documented as robust SD-OCT based imaging biomarkers for predicting visual outcome in eyes with center involving diabetic macular edema, retinitis pigmentosa and other inherited macular disorders. Hyperreflectivity of the external limiting membrane and can serve as possible transient biomarker of early Stargardt disease. Novel imaging parameter ‘parallelism’ serves as a potential biomarker to prognosticate visual outcome in diabetic macular edema and ERM. Three dimensional imaging along with segmentation techniques provided comprehensive evaluation of the surface topography as well as foveal and extrafoveal anatomical configuration of vitreomacular interface disorders.

OCT technology is changing very quickly and throughout this review there are some of the multiple possibilities that OCT based imaging biomarkers will be more useful in the near future for diagnosis, prognosticating disease progression and as endpoint in clinical trials.

## Abbreviations

SD-OCT: spectral domain optical coherence tomography
RNFL: retinal nerve fiber layer
CST: central subfield thickness
ONL: outer nuclear layer
GCL: ganglion cell layer
CAT: cube average thickness
AMD: age related macular degeneration
nAMD: neovascular age related macular degeneration
HF: hyperreflective foci
RPEDC: retinal pigment epithelium-drusen complex
DR: diabetic retinopathy
ELM: external limiting membrane band
EZ: ellipsoid zone
ODR: optical density ratio
RPE: retinal pigment epithelium
PCV: polypoidal choroidal vasculopathy
ERM: epiretinal membrane
VMI: vitreomacular interface
VMA: vitreomacular adhesion
VMT: vitreomacular traction
FTMH: full thickness macular hole
